# p27 specifically decreases in squamous carcinoma, and mediates NNK‐induced transformation of human bronchial epithelial cells

**DOI:** 10.1111/jcmm.18577

**Published:** 2024-08-04

**Authors:** Minggang Peng, Hao Meng, Jingjing Wang, Mengxin Guo, Tengda Li, Xiaohui Qian, Ruifan Chen, Honglei Jin, Chuanshu Huang

**Affiliations:** ^1^ Key Laboratory of Medicine, Ministry of Education, School of Laboratory Medicine and Life Sciences Wenzhou Medical University Wenzhou Zhejiang China; ^2^ Department of Obstetrics and Gynecology, Union Hospital, Tongji Medical College Huazhong University of Science and Technology Wuhan Hubei China; ^3^ Oujiang Laboratory (Zhejiang Lab for Regenerative Medicine, Vision and Brain Health) Wenzhou Zhejiang China

**Keywords:** lung carcinogenesis, miR‐494, NNK, p27, STAT3

## Abstract

Lung cancer remains the leading cause of cancer‐related deaths, with cigarette smoking being the most critical factor, linked to nearly 90% of lung cancer cases. NNK, a highly carcinogenic nitrosamine found in tobacco, is implicated in the lung cancer‐causing effects of cigarette smoke. Although NNK is known to mutate or activate certain oncogenes, its potential interaction with p27 in modulating these carcinogenic effects is currently unexplored. Recent studies have identified specific downregulation of p27 in human squamous cell carcinoma, in contrast to adenocarcinoma. Additionally, exposure to NNK significantly suppresses p27 expression in human bronchial epithelial cells. Subsequent studies indicates that the downregulation of p27 is pivotal in NNK‐induced cell transformation. Mechanistic investigations have shown that reduced p27 expression leads to increased level of ITCH, which facilitates the degradation of Jun B protein. This degradation in turn, augments miR‐494 expression and its direct regulation of JAK1 mRNA stability and protein expression, ultimately activating STAT3 and driving cell transformation. In summary, our findings reveal that: (1) the downregulation of p27 increases Jun B expression by upregulating Jun B E3 ligase ITCH, which then boosts miR‐494 transcription; (2) Elevated miR‐494 directly binds to 3′‐UTR of JAK1 mRNA, enhancing its stability and protein expression; and (3) The JAK1/STAT3 pathway is a downstream effector of p27, mediating the oncogenic effect of NNK in lung cancer. These findings provide significant insight into understanding the participation of mechanisms underlying p27 inhibition of NNK induced lung squamous cell carcinogenic effect.

## 1 INTRODUCTION

Lung cancer remains the leading cause of cancer‐related mortality worldwide. In 2021, the United States alone reported approximately 235,760 new cases and 131,880 deaths, significantly outnumbering other cancers.^1^ Cigarette smoking is identified as the primary risk factor, contributing to 90% of lung cancer cases.^2^ Lung squamous cell carcinoma (LUSC), a subtype of non‐small cell lung cancer (NSCLC), comprises about 40% of lung cancer cases, with its occurrence linked to age and the extent of tobacco smoke exposure.^3^ LUSC is known for its poor clinical prognosis and the limited availability of targeted therapies compared to lung adenocarcinoma (LUAD).^4^, ^5^ The biomarkers associated with LUSC and precise therapeutic targets remain underexplored.

Tobacco smoke contains over 60 carcinogens, including potent substances like polycyclic hydrocarbons and the nitrosamines. Nitrosamine 4‐(methylnitrosamino)‐1‐(3‐pyridyl)‐1‐butanone (NNK), a nicotine derivative, is one of the most carcinogenic nitrosamines. It has shown to induce lung tumours, including squamous cell carcinoma, adenocarcinoma and adenosquamous carcinoma.^6^, ^7^ NNK acts as a procarcinogen that can be metabolized and activated in the lung and liver.^8^ While NNK exposure has been linked to mutations/activations in oncogenes, such as K‐ras, p53 or the EGFR,^9^ its impact on the tumour suppressor CDKN1B (p27) is yet to be established. p27 is a known cyclin‐dependent kinase inhibitor involved in regulating cell cycle, apoptosis, tumorigenesis and tumour invasion.^10^ Our studies revealed that p27 downregulation mediates NNK‐induced bronchial epithelial cell transformation via promoting miR‐494 expression. MiRNAs are evolutionarily conserved, single stranded, non‐protein‐coding RNAs that functioning as post‐transcriptional gene regulators. They typically inhibit targeted gene expression through repressing messenger RNA (mRNA) translation or influencing mRNA degradation^11^ by complementary binding to the 3′ untranslated region (3′‐UTR) of their target mRNAs.^12^ Our group demonstrated that miR‐494 inhibition is crucial for stabilizing PTEN mRNA via tumour suppressor p100, thereby acting as an oncogene.^13^ Here, we found that miR‐494 is remarkably induced by NNK and highly expressed in LUSC. Janus kinases (JAKs) is a family of intracellular and non‐receptor tyrosine kinase that transduce cytokine‐mediated signals via its binding specifically to intracellular domains of cytokine receptors. They are activated through receptor multimerization upon ligand binding, leading to phosphorylation of STATs, which then translocate to the nucleus and regulate gene expression.^14^ The JAK/STAT3 pathway is known to promote lung cancer progression, with overexpression of p‐STAT3 correlating with poor prognosis.^15^, ^16^ Our studies indicate that NNK exposure activate the JAK1/STAT3 pathway, mediating its oncogenic effects of NNK via inhibiting p27 expression.

p27 has been identified as a tumour suppressor for nearly two decades, playing roles in cell cycle control, differentiation, senescence and apoptosis.^17^ Our research extends the understanding of p27 tumour suppressive function by revealing its inhibitory effect on the JAK1/STAT3 pathway. Our findings demonstrate that knockout or knockdown of p27 leads to increased JAK1 protein expression, and enhanced phosphorylation of STAT3 at site Y705. This inhibition of JAK1 by p27 is dependent on miR‐494, which binds to 3′‐UTR of JAK1 mRNA, influencing its degradation.

## 2 MATERIALS AND METHODS

### 2.1 Patients and tumour sample preparation

Twelve pairs of lung squamous carcinoma, 11 pairs of lung adenocarcinoma and their adjacent non‐tumour tissue specimens were obtained from The First Affiliated Hospital of Wenzhou Medical University (Wenzhou, China) upon the informed consent. Inclusion and exclusion criteria: Lung carcinoma was histopathological diagnosed while adjacent non‐tumour tissue specimens were taken from a standard distance (3 cm) from the margin of resected neoplastic tissues of patients with tumours who ensured surgical lung ablation. Only pairs of tissues were included, otherwise, it was excluded from the study. The cancer cases were randomized selected. This study was compliant with the Declaration of Helsinki Guidelines and was approved by the Medical Ethics Committee of Wenzhou Medical University. Based on the cut‐off value of CDKN1B (p27 protein corresponding gene), patients diagnosed with LUSC or LUAD were divided into two groups. The survival analysis was performed on the human protein atlas portal (HPA) (https://www.proteinatlas.org/). Immunohistochemistry results are also from HPA. The proteome or miRNA files of LUSC and LUAD were collected from the cancer genome atlas program (TCGA) (https://portal.gdc.cancer.gov/).

### 2.2 Reagents, antibodies and plasmids

The TRIzol reagent and SuperScript™ First‐Strand Synthesis system was bought from Invitrogen (Grand Island, NY). Chemicals of Actinomycin D (Act D), MG132, CHX and doxycycline were purchased from Santa Cruz (Dallas, TX). The specific chemical NNK was purchased from Toronto Research Chemicals (Toronto, Canada). The Dual Luciferase Assay kit was purchased from Promega (Madison, WI). Antibodies specific against p‐STAT3 Y705, STAT3, JAK1, AUF1, HuR, STAT5, SOX‐2, p53, ITCH, Elk‐1, Myc‐tag and HA were purchased from Cell Signalling (Beverly, MA). Antibodies specific for p27, Jun B, NCL and β‐Actin were from Santa Cruz (Dallas, TX). Specific antibody against GAPDH was from Genetex (Invine, CA). The shRNAs that specifically target mouse p27, Jak1, NCL with their nonsense controls and miRNA‐494 inhibitor were purchased from Open Biosystems (GE, Pittsburgh, PA). The Jun B expression plasmid was kindly gifted from Dr. Bravo (University of Pennsylvania, Philadelphia). The plasmid GFP‐p27 and HA‐STAT3 Y705F was described in our previous studies.^17^, ^18^ The plasmid Myc‐ITCH (WT) and Myc‐ITCH (MUT) were purchased from Addgene (#11427, #11428). The plasmid pTRE‐GFP‐p27 was constructed into pTRE‐tight vector using the primers: forward 5′‐GGG GTA CCC GCC ACC ATG GTG AGC AAG‐3′, reverse 5′‐GGT GGA TCC TTA CGT TTG ACG TCT‐3′. The plasmid containing luciferase reporter under control of mouse miRNA‐494 promoter were constructed into PGL3‐Basic vector using the primers: forward 5′‐CCG CTC GAG GTG TCT GAC AAT AAC TCT TGT GTG G‐3′, reverse 5′‐CCC AAG CTT TTC AAA CAC AGA AAC CCC TC‐3′.

### 2.3 Cell lines and transfection

Immortalized wild‐type p27(p27+/+) and p27‐deficient (p27−/−(Δ51)) mouse embryonic fibroblasts (MEFs) were described in previous studies^18^ and cultured in DMEM with 10% FBS. Beas‐2B and BEP2D cells were described in our previous studies^19^ and cultured in DMEM with 10% FBS, LHC‐8 medium (Gibco, United States), respectively. Normal mouse epidermal Cl41 cells, was cultured in MEM with 5% FBS. Cell transfections were performed with PolyJet™ DNA in Vitro Transfection Reagent (SignaGen Laboratories, Rockville, MD), according to the manufacturer's instructions. For stable transfection, MEF cells were subjected to selection with hygromycin B (200 μg/mL) or puromycin (2.0 μg/mL) depending on the different antibiotic resistance of plasmids transfected.

### 2.4 Western blot analysis

Whole cell extracts were prepared with the cell lysis buffer (10 mM Tris–HCl, pH 7.4, 1% SDS and 1 mM Na_3_VO_4_) as described in our previous studies.^18^ Protein extracts were subjected to western blot with the indicated primary antibodies, and probed with the AP‐conjugated secondary antibody together with the enhanced chemifluorescence system. The images were acquired by scanning with the PhosphorImager Typhoon FLA 7000 (GE, Pittsburgh, PA).

### 2.5 Luciferase reporter assay

The miR‐494 promoter luciferase reporter, Jak1 promoter luciferase reporter and pRL‐TK, were transfected into p27+/+, p27−/−(Δ51) or other related transfectants. Twenty‐four hours after the transfection, luciferase activity was determined using the Dual Luciferase Assay system kit purchased from Promega (Madison, WI,). The results were normalized by internal TK signal.

### 2.6 ChIP assay

The EZ‐ChIP kit (Millipore Technologies, Billerica, Massachusetts) was used to carry out the ChIP assay according to the manufacturer's instructions and as described previously.^20^ Briefly, p27+/+ cells were treated with 1% formaldehyde for 10 min at room temperature. Cells were then pelleted, resuspended in lysis buffer and sonicated to generate 200–400 bp chromatin DNA fragments. After centrifugation (13,000 *g* for 10 min at 4°C), a 10‐fold dilution of the supernatants were incubated with an anti‐Jun B antibody or the control rabbit IgG at 4°C overnight. The immune complex was captured with Protein G–agarose‐saturated beads with salmon sperm DNA and then eluted with elution buffer. The reverse cross‐linking of protein–DNA complexes to free DNA was conducted by incubating at 65°C overnight. The DNA was extracted and subjected to PCR analysis. To specifically amplify the region containing the Jun B‐binding sites on the mouse miR‐494 promoter, PCR was performed with the following pair of primers: 5′‐AAT AAC TCT TGT GTG GCA‐3′, and 5′‐ATA TAG CGT GTG CTC TCT‐3′.

### 2.7 RT‐PCR

Total RNA was extracted using the TRIzol reagent (Invitrogen, Grand Island, NY) as described in the manufacturer's instructions. Five microgram of total RNA was used for first‐strand cDNA synthesis with oligdT primer by SuperScript™ IV First‐Strand Synthesis System (Invitrogen, Grand Island, NY). Specific primers (Invitrogen, Grand Island, NY) used for PCR amplification are exhibited in Table 1.

**TABLE 1:** Specific primers used for PCR amplification.

mouse jak1:	Forward 5′‐GGC TAC CTT GGA AAC TTT GAC‐3′
	Reverse 5′‐GAA AAA TTG TTC CAC TCT TCC C‐3′
mouse junb:	Forward 5′‐GCA GCT ACT TTT CGG GTC AG‐3′
	Reverse 5′‐ATG TGG GAG GTA GCT GAT GG‐3′
mouse gapdh:	Forward 5′‐TGC AGT GGC AAA GTG GAG ATT‐3′
	Reverse 5′‐TTT TGG CTC CAC CCT TCA AGT‐3′
mouse β‐actin:	Forward 5′‐GAC GAT ATT GCC GCA CT‐3′
	Reverse 5′‐GAT ACC ACG CTT GCT CTG AG‐3′

### 2.8 Quantitative RT‐PCR

The cells used for total RNA extraction were using miRNeasy Mini Kit (QIAGEN, Valencia, CA). Analysis of miR‐17, miR‐20a, miR‐20b, miR‐93, miR‐106b and miR‐494 expression were carried out using the miScript PCR system (QIAGEN, Valencia, CA) by 7900HT Fast Real‐time PCR system (Applied Biosystems, Foster City, CA). The primers for real‐time PCR assay and internal control U6 were purchased from Invitrogen (Grand Island, NY). The initial activation was performed at 95°C for 15 min, followed by 40 cycles (denaturation at 95°C for 15 s, annealing at 55°C for 30 s and extension at 72°C for 30 s).

### 2.9 Anchorage‐independent growth assay

The anchorage‐independent growth ability of human bronchial epithelial cell line BEP2D and its transfectants followed NNK exposure and doxycycline treatment was determined in soft agar. Briefly, 3 mL of 0.5% agar in basic LHC‐8 medium was layered onto each well of 6‐well tissue culture plates. One millilitre of BEP2D cells or their transfectants (1 × 10^4^) mixed with 0.5% agar in LHC‐8 (1:3) were then layered on top of the basal layer in each well of 6‐well tissue culture plates. Plates were incubated at 37°C in 5% CO_2_ for 3 weeks, and the colonies with more than 32 cells were scored and are presented as colonies/10^4^ cells.

### 2.10 Statistical analysis

The student's t‐test was used to determine the significance between treated and untreated group. The results are expressed as mean ± SD from at least three independent experiments. *p* < 0.05 was considered to be a significant difference between compared groups.

## 3 RESULTS

### 3.1 Downregulation of p27 was observed in human LUSC and NNK‐exposed human bronchial epithelial cells (HBECs), and contributed to NNK‐induced transformation of HBECs

**FIGURE 1:**
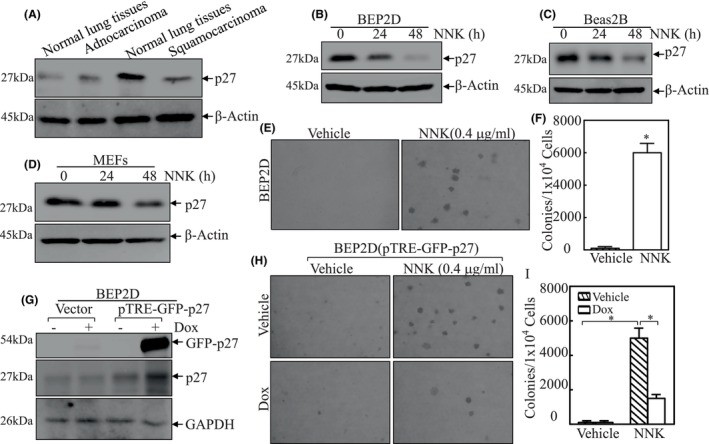
Downregulation of p27 contributed to NNK‐induced transformation of HBECs. (A) Western blot (WB) was used to evaluate p27 protein expression in the mixed human lung cancer tissues (*n* = 11 for adenocarcinoma and *n* = 12 for squamous carcinoma). β‐Actin was used as protein loading control. (B–D) Western blot (WB) was used to determine p27 protein expression in the NNK‐exposed BEP2D cells (B), Beas2B cells (C) or MEF cells (D) as indicated. β‐Actin was used as protein loading control. (E and F) The anchorage‐independent growth abilities of BEP2D with or without NNK exposure were determined in soft agar assay. The results were presented as colonies/1 × 10^4^ cells. The symbol (*) indicates a significant increase in comparison to vehicle‐treated cells (*p* < 0.05). (G) Stable inducible transfectants of BEP2D(Vector) versus BEP2D(pTRE‐GFP‐p27) were treated with or without doxycycline and the cell extracts were subjected to western blot for identification of GFP‐p27 expression. GAPDH was used as protein loading control. (H and I) The BEP2D(pTRE‐GFP‐p27) were first treated with or without doxycycline same as described in (G), and the cells were then subjected to anchorage‐independent growth assay in the presented with either control vehicle or NNK. The results were presented as colonies/1 × 10^4^ cells. The symbol (*) indicates a significant difference (*p* < 0.05).

p27 is recognized as a tumour suppressor due to its role as a cyclin‐dependent kinase inhibitor for regulating the cell cycle and growth. Notably, its downregulation has been observed in a various human cancer tissues, including non‐small cell lung carcinoma.^21^ To investigate the differential expression of p27 in different types of human lung cancers, we compared p27 protein expression levels in human squamous carcinoma and adenocarcinoma. Given the limited availability of lung carcinoma tissues from each patient were obtained from each patient, we standardized the approach by collecting an equal amount of total protein from each patient and combining them for western blot analysis. The results showed a significant decrease in p27 levels in lung squamous carcinoma tissues compared to normal human lung tissues, while such downregulation was not observed in human adenocarcinoma tissues (Figure 1A). This intriguing finding is further supported by our analysis of public databases, which show a correlation between CDKN1B mRNA and p27 protein expression. We observed that the survival probability of LUSC patients with high p27 mRNA levels was significantly greater than those with low expression levels, a trend not seen in LUAD patients (Figure S1A). Additionally, TCGA database analysis indicated that p27 protein levels were significantly lower in LUSC patients compared to those with LUAD (Figure S1B). Histochemical analysis confirmed lower p27 expression in LUSC tissue samples relative to LUAD tissues (Figure S1C).

To further explore the role of p27 in lung squamous carcinoma carcinogenesis, we examined whether p27 is downregulated in normal human bronchial epithelial cells (NHBECs) exposed to NNK. Our findings (Figure 1B–D) show that NNK significantly reduce p27 protein expression in BEP2D, Beas‐2B and MEF cells, suggesting involvement of p27 in NNK‐exposed HBECs. Furthermore, we evaluated the effect of NNK on anchorage‐independent growth abilities. NNK exposure was found to markedly enhance the anchorage‐independent growth capabilities of BEP2D cells (Figure 1E,F), indicating that NNK exposure induce BEP2D cell transformation. Consequently, we transfected BEP2D cells with pTRE‐GFP‐p27. These transfectants expressed GFP‐p27 ectopically upon doxycycline (Dox) treatment (Figure 1G). Soft agar assays showed that the inducible overexpression of p27 significantly inhibited cell transformation compared to scrambled control vector transfectants (Figure 1H,I). These comprehensive results strongly suggest that p27 act as a negative regulator in NNK‐induced BEP2D cell transformation.

### 3.2 p27 attenuated phosphorylation of STAT3 on Y705 through inhibition of JAK1 expression

**FIGURE 2:**
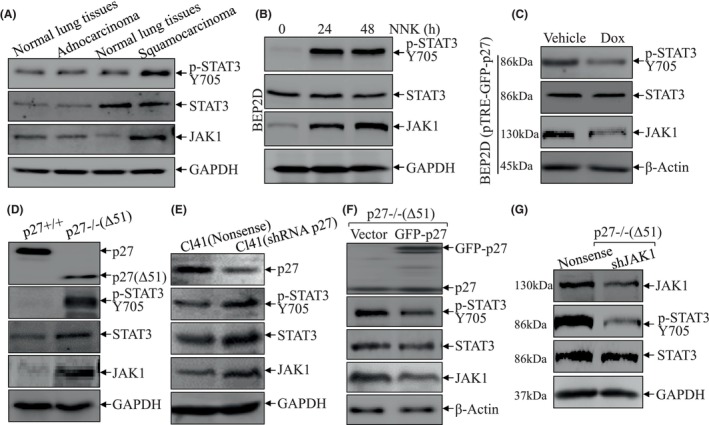
p27 attenuated phosphorylation of STAT3 on Y705 through inhibition of JAK1 expression. (A) The protein expression of JAK1, STAT3 and p‐STAT3 at Y705 in the indicated human lung cancer tissues were evaluated using western blot. GAPDH was used as protein loading control. (B) BEP2D cells were exposed to NNK as indicated and cell extracts were subjected to western blot (WB) to detect the protein expression levels of STAT3, p‐STAT3 at Y705 and JAK1. GAPDH was used as protein loading control. (C) Cell extracts from BEP2D(pTRE‐GFP‐p27) cells treated with or without doxycycline exposure were subjected to western blot for determination of STAT3, p‐STAT3 at Y705 and JAK1. β‐Actin was used as protein loading control. (D–F) The cell extracts as indicated were subjected to western blot to detect the protein levels of STAT3, p‐STAT3 and JAK1 in p27+/+ versus p27−/−(Δ51) cells (D), Cl41(Vector) versus Cl41(shp27) cells (E) and p27−/−(Δ51) versus p27−/−(Δ51/GFP‐p27) cells (F); GAPDH or β‐Actin were used as protein loading control. (G) p27−/−(Δ51) cells were stably transfected with shRNA JAK1 and the cell extracts were subjected to western blot to define the protein expression levels of JAK1 STAT3 and p‐STAT3 at Y705; GAPDH was used as protein loading control.

JAK/STAT3 pathway is widely reported to participate in the carcinogenesis of lung cancer,^22^, ^23^, ^24^ the STAT3 and its kinase JAK1 protein expressions were firstly evaluated in the same human lung carcinoma tissues as described in Figure 1A. As shown in Figure 2A, there is a remarkable increase in STAT3 phosphorylation level at site Y705 in human squamous carcinoma tissues compared to their adjacent normal lung tissues, whereas the total STAT3 protein expression showed no distinguishable difference. Consistently, JAK1 protein level also exhibited a dramatic increase in squamous carcinoma tissues compared to normal controls (Figure 2A). In contrast to squamous carcinoma, neither STAT3 nor JAK1 presented any observable differences in adenocarcinoma tissues. These results reveal the possibility that JAK1/STAT3 pathway might be a p27 downstream effector in lung squamous carcinoma development but not in adenocarcinoma. Therefore, we tested the effects of NNK exposure on JAK1/STAT3 axis in BEP2D cells. As showed in Figure 2B, upon NNK exposure, STAT3 phosphorylation at site Y705 and JAK1 protein expression were markedly induced, while the total STAT3 protein showed no effect in BEP2D cells. To further determine the regulatory effect of p27 on JAK1/STAT3 activation, inducible GFP‐p27 expression construct was stably transfected into BEP2D cells. The results shown in Figure 2C exhibited that p27 overexpression significantly attenuated JAK1 expression as well as STAT3 phosphorylation at Y705 with minor effects on STAT3 expression, demonstrating the negative regulation of p27 on JAK1/STAT3 pathway. p27−/−(Δ51) cells, which harbouring a disrupted p27 gene as described in our previous studies,^10^ and p27+/+ cells were also subjected to investigate the effects of p27 on JAK1/STAT3 pathway. The results validated that p27 deletion evidently promoted JAK1 protein expression and STAT3 phosphorylation at Y705, while it slightly enhanced STAT3 protein expression (Figure 2D). This notion was further supported by the data obtained from knockdown of p27 in mouse epithelial Cl41 cells (Figure 2E). Consistently, reintroduction of GFP‐p27 into p27−/−(Δ51) cells inhibited JAK1 protein and STAT3 phosphorylation at Y705 (Figure 2F). To evaluate the regulatory effects of JAK1 on STAT3, JAK1 shRNA was employed to knockdown JAK1 in p27−/−(Δ51) cells. As shown in Figure 2G, knockdown of JAK1 dramatically inhibited STAT3 phosphorylation at Y705 with undistinguishable effects on total STAT3 protein expression. Collectively, our results suggest that inhibition of JAK1/STAT3 pathway by p27 is associated with its tumour suppressive effect in lung carcinogenesis. It is worth noting that p27/JAK1/STAT3 axis is crucial in lung squamous carcinoma carcinogenesis but not adenocarcinoma.

### 3.3 miR‐494 induction due to p27 downregulation resulted in Jak1 mRNA stabilization and consequently led to transformation of lung epithelial cells upon NNK exposure

**FIGURE 3:**
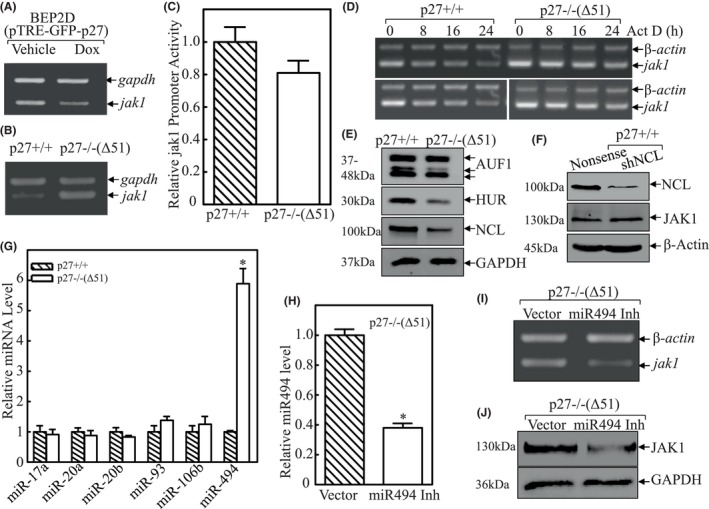
miR‐494 induction mediated Jak1 mRNA stabilization and transformation of lung epithelial cells upon NNK exposure. (A) RT‐PCR was applied to compare the mRNA levels of jak1 in BEP2D(pTRE‐GFP‐p27) cells treated with or without doxycycline exposure; gapdh was used as internal control. (B) RT‐PCR was applied to compare the mRNA levels of Jak1 between p27+/+ and p27−/−(Δ51) cells; gapdh was used as internal control. (C) Mouse jak1 promoter‐driven luciferase reporter was co‐transfected together with pRL‐TK into p27+/+ and p27−/−(Δ51) cells, respectively. Twenty‐four hours post transfection, the transfectants were extracted to evaluate the luciferase activity. TK was used as internal control. The results were presented as relative jak1 promoter activity, and each bar indicates a mean ± SD from three independent assays. (D) p27+/+ and p27−/−(Δ51) cells were subjected to mRNA degradation assay in the presence of Act D for the indicated time points. The cell extracts were then subjected to RT‐PCR to analyse jak1 mRNA degradation rates between the indicated cells. β‐Actin was used as loading control. (E and F) The indicated cell extracted were subjected to western blot to evaluate the protein expression levels of AUF1, HUR, NCL and JAK1; GAPDH was used as protein loading control. (G) The indicated cells were employed to determine the expression levels of miR‐17a, miR‐20a, miR‐20b, miR‐93, miR‐106b and miR‐494 by real‐time PCR. The results were normalized to *Rnu6*. The symbol (*) indicates a significant increase in comparison to p27+/+ cells (*p* < 0.05). (H) Real‐time PCR was employed to identify the expression levels of miR‐494 in p27−/−(Δ51) cells that were stably transfected with miR‐494 inhibitor (miR‐494 Inh). (I) RT‐PCR was applied to compare the mRNA levels of jak1 between p27−/−(Δ51) and p27−/−(Δ51/miR‐494 inhibitor) cells; β‐Actin was used as internal control. (J) Western blot was used to evaluate the protein expression levels of JAK1 in p27−/−(Δ51/Vector) and p27−/−(Δ51/miR‐494 inhibitor) cells; GAPDH was used as protein loading control.

To elucidate the mechanism underlying p27 inhibition of JAK1 expression, Jak1 mRNA was first evaluated in BEP2D (pTRE‐GFP‐p27) cells treated with or without doxycycline. As observed in Figure 3A, Jak1 mRNA was significantly attenuated in p27 overexpressed BEP2D cells. The similar effect of p27 on jak1 mRNA level was consistently observed in p27+/+ versus p27−/−(Δ51) cells (Figure 3B). Thus, we next determined whether the inhibition of Jak1 mRNA expression by p27 occurred at mRNA transcription level. The Jak1 promoter‐driven luciferase reporter was transfected into p27+/+ and p27−/−(Δ51) cells (Figure 3C), respectively. The results showed no significant difference of Jak1 promoter‐driven transcriptional activities between p27+/+ and p27−/−(Δ51) cells, excluding the possibility that p27 regulates Jak1 transcription. Next, Act D was used to block new mRNA transcription in its treated cells to evaluate Jak1 mRNA degradation rates. The results shown in Figure 3D indicate that Jak1 mRNA degradation in p27−/−(Δ51) cells is retarded in comparison to p27+/+ cells, providing strong evidence that p27 inhibits Jak1 mRNA expression by promoting its degradation. Given that AUF1, HUR and NCL proteins are three well‐known RNA binding proteins that can bind and mediate targeted mRNA stability,^25^, ^26^ we evaluated their potential involvements in modulating Jak1 mRNA stability in p27+/+ and p27−/−(Δ51) cells. We found that expression of AUF1, HUR and NCL were all reduced in p27−/−(Δ51) cells in comparison to p27+/+ cells (Figure 3E). As reported, AUF1 and HUR generally stabilize its targeted mRNAs, NCL were analysed for its potential contribution in p27 regulation of JAK1 expression. The results in Figure 3F exhibited that knockdown of NCL in p27+/+ cells did not alter JAK1 protein expression, excluding NCL regulation of JAK1 mRNA stability.

It has been reported that miRNA can also bind to 3′‐UTR of target gene mRNA to modulate its mRNA stability.^27^ Thus, the bioinformatics programs, TFSearch and PROMO, were used to seek for potential miRNAs involved in the regulation of JAK1 mRNA stability. According to the result, the 3′‐UTR region of Jak1 mRNA contains multiple miRNA binding sites, including miR‐17a, miR‐20a, miR‐20b, miR‐93, miR‐106b and miR‐494 (Table 2). To define the specific miRNA that might be involved in stabilization of Jak1 mRNA, the listed miRNA expression levels were determined in p27+/+ and p27−/−(Δ51) cells. As shown in Figure 3G, the expression levels of miR‐17a, miR‐20a, miR‐20b, miR‐93 and miR‐106b all exhibited no significant difference between these two cells, while miR‐494 was remarkably elevated in p27−/−(Δ51) cells in comparison to p27+/+ cells, strongly indicating that miR‐494 may participate in the regulation of Jak1 mRNA stability. This notion was supported by the results obtained from overexpression of miR‐494 inhibitor in p27−/−(Δ51) cells (Figure 3H–J). The inhibition of miR‐494 led to a profound decrease of Jak1 mRNA expression as well as JAK1 protein expression, illustrating that miR‐494 is responsible for p27‐mediated Jak1 mRNA stability in MEF cells.

**TABLE 2:** Sequence alignments of potential miRNAs with JAK1 3′UTR.

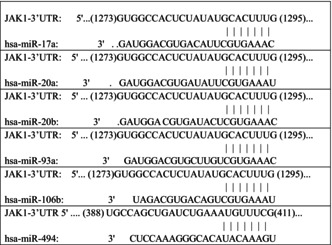

**FIGURE 4:**
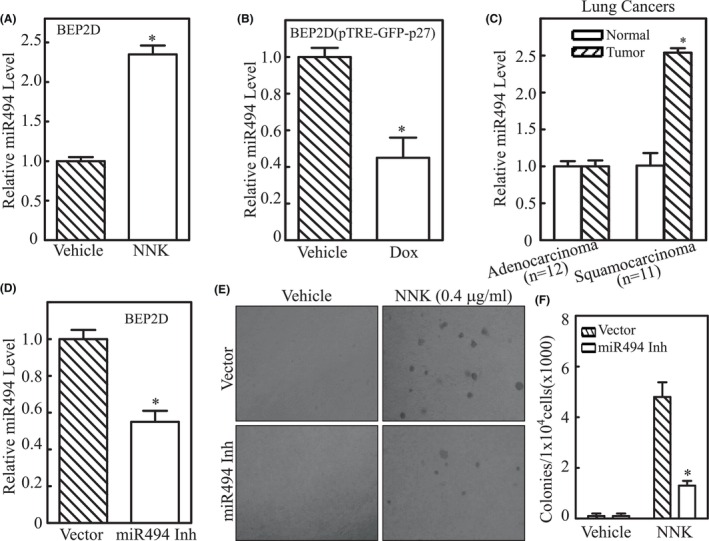
miR‐494 upregulation due to p27 deletion promoted NNK‐induced transformation of lung epithelial cells upon NNK exposure. (A and B) Real‐time PCR was employed to determine the expression levels of miR‐494 in BEP2D cells treated with or without NNK for 24 h (A), or BEP2D(pTRE‐GFP‐p27) cells treated with or without doxycycline exposure (B), respectively. The results were normalized to *Rnu6*, and the symbol (*) indicates a significant difference (*p* < 0.05). (C) Real‐time PCR was used to evaluate the expression level of miR‐494 in the human lung cancer tissues in comparison to their paired non‐cancer tissues; the symbol (*) indicates a significant increase (*p* < 0.05). (D) Real‐time PCR was employed to determine the expression levels of miR‐494 in BEP2D cells that were stably transfected with miR‐494 inhibitor. (E and F) The anchorage‐independent growth ability of BEP2D(Vector) and BEP2D (miR‐494 inhibitor) cells upon exposure to NNK or vehicle control were evaluated in soft agar assay. The results were presented as colonies/1 × 10^4^ cells. The symbol (*) indicates a significant inhibition (*p* < 0.05).

Therefore, we next determine the role of miR‐494 in NNK‐induced transformation in NHBECs. As expected, the miR‐494 expression in BEP2D cells markedly induced upon NNK exposure (Figure 4A), while overexpression of GFP‐p27 dramatically inhibited miR‐494 expression (Figure 4B). We then evaluated miR‐494 expression in human lung cancer tissues that is abovementioned. A significant higher expression of miR‐494 was observed in lung squamous carcinoma tissues compared to normal controls while no noticeable difference was exhibited between adenocarcinoma and corresponding controls (Figure 4C). This finding was strongly supported by the results obtained from the TCGA database (*p* < 0.0001, Figure S1D). miR‐494 level in T1 stage was significantly lower than that of T2 stage, which was significantly lower than that of T3 stage (*p* < 0.05, Figure S1E). To further verify the role of miR‐494 in promoting lung epithelial cell transformation, we stably transfected miR‐494 inhibitor into BEP2D cells. The cells were identified as shown in Figure 4D, and employed in soft agar assays. The results indicated that the BEP2D cell transformation induced by NNK was inhibited after miR‐494 was repressed as compared to their scrambled control vector transfectants (Figure 4E,F). Taken together, we concluded that reduction of miR‐494 was critical for p27‐mediated inhibition of lung epithelial cell transformation upon NNK exposure.

### 3.4 p27 promoted Jun B expression, which binds directly to miR‐494 promoter region and inhibited miR‐494 transcription

**FIGURE 5:**
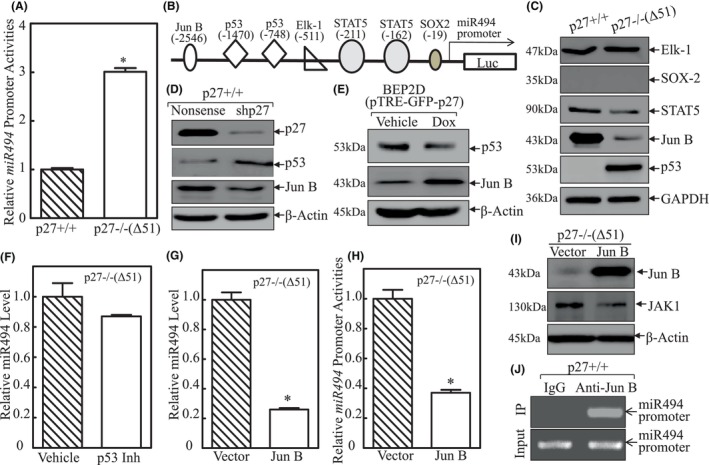
p27 promoted Jun B expression, which binds directly to miR‐494 promoter region and inhibited miR‐494 transcription. (A) miR‐494 promoter‐driven luciferase reporter was co‐transfected together with pRL‐TK into p27+/+ and p27−/−(Δ51) cells, respectively. Luciferase activity was evaluated and the results were presented as relative miR‐494 promoter activity, and each bar indicates a mean ± SD from three independent assays. The symbol (*) indicates a significant increase in comparison to p27+/+ cells (*p* < 0.05). (B) The potential binding sites for transcription factors in the miR‐494 promoter region. (C) Western blot was employed to determine the protein expressions of ELK1, SOX‐2, STAT5, p53 and Jun B in p27+/+ versus p27−/−(Δ51) cells; GAPDH was used as protein loading control. (D and E) Western blot was employed to evaluate the protein expressions of p53 and Jun B in p27+/+ versus p27−/−(Δ51) cells (D), and BEP2D(pTRE‐GFP‐p27) cells treated with or without doxycycline exposure (E), respectively. β‐Actin was used as protein loading control. (F and G) Real‐time PCR was employed to determine the expression levels of miR‐494 in p27−/−(Δ51) cells stably transfected with p53 inhibitor (F), or Jun B (G). The symbol (*) indicates a significant inhibition (*p* < 0.05). (H) miR‐494 promoter‐driven luciferase reporter was co‐transfected together with pRL‐TK into p27−/−(Δ51/Vector) and p27−/−(Δ51/Jun B) cells. Luciferase activity was evaluated and the results were presented as relative miR‐494 promoter activity; The symbol (*) indicates a significant inhibition in comparison to Vector transfectant (*p* < 0.05). (I) Western blot (WB) was used to evaluate the effect of Jun B overexpression on JAK1 protein expression in p27−/−(Δ51/Vector) versus p27−/−(Δ51/JUNB) cells; β‐Actin was used as protein loading control. (J) ChIP assay was employed to determine Jun B binding to the miR‐494 promoter region using anti‐Jun B antibody.

To clarify the role of miR‐494 in p27 inhibition of JAK1/STAT3 expressions and functions, we investigated the underlying mechanisms revealing p27 regulation of miR494 expression. As shown in Figure 5B, the full length of the miR‐494 promoter‐driven luciferase reporter was constructed and transfected into p27+/+ and p27−/−(Δ51) cells to verify whether p27 modulates miR‐494 at transcriptional level. The results in Figure 5A showed that miR‐494 promoter activity was significantly increased in p27−/−(Δ51) cells as compared to that in p27+/+ cells. This result revealed that p27 might inhibit miR‐494 transcription. Therefore, the transcription factors that might potentially bind to the promoter region of miR‐494 were determined in p27+/+ and p27−/−(Δ51) cells (Figure 5C). Based on the results, STAT5 and Jun B were impaired in p27−/−(Δ51) cells, whereas p53 expression was increased in p27−/−(Δ51) cells, and ELK1 was comparable between the two cells (Figure 5C). Given that STAT5 has been reported to promote its regulated gene transcription,^28^ Jun B and p53 were further analysed for their potential effects on miR‐494 expression. As shown in Figure 5D,E, knockdown of p27 increased p53 expression and decreased Jun B expression, while ectopic expressed GFP‐p27 inhibited p53 and promoted Jun B expression. p53 inhibitor was then transfected in p27−/−(Δ51) cells with no significant effect on miR‐494 expression (Figure 5F); while overexpression of Jun B led to a dramatic decrease in miR‐494 expression in p27−/−(Δ51) cells (Figure 5G), indicating that Jun B, but not p53, plays an inhibitory role in miR‐494 transcription. This notion is further supported by the results showing that ectopic expression of Jun B increased miR‐494 promoter transcriptional activity (Figure 5H). Moreover, the results obtained in Figure 5I reveal that Jun B could inhibit the downstream JAK1 protein expression. In addition, the results from the CHIP assay suggest that Jun B could directly bind to the miR‐494 promoter region (Figure 5J). Taken together, we have clearly revealed the mechanisms underlying p27 regulation of JAK1/STAT3 pathway. All the evidence in Figure 5 support the notion that p27 may promote Jun B expression, which directly binds to miR‐494 promoter region, and attenuating miR‐494 transcription to suppress JAK1 protein expression.

### 3.5 p27 inhibited expression of ITCH, a E3 ligase of Jun B, by which led to Jun B protein accumulation

**FIGURE 6:**
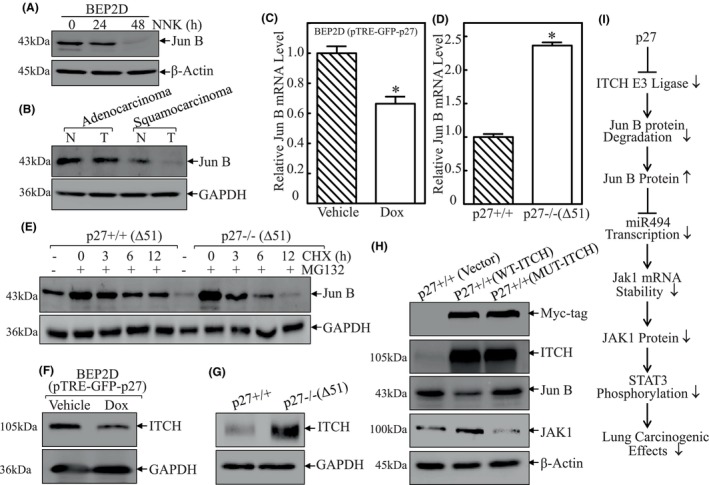
p27 inhibited expression of ITCH, a E3 ligase of Jun B, by which led to Jun B protein accumulation. (A) Western blot (WB) was employed to detect the protein expression of Jun B in BEP2D cells exposed to NNK; β‐Actin was used as protein loading control. (B) Western blot was employed to evaluate the protein levels of Jun B in human lung cancer tissues; GAPDH was used as protein loading control. (C and D) Real‐time PCR was employed to evaluate the expression levels of Jun B mRNA in BEP2D(pTRE‐GFP‐p27) cells treated with or without doxycycline exposure (C), and p27+/+ versus p27−/−(Δ51) cells (D), respectively. The symbol (*) indicates a significant difference (*p* < 0.05). (E) p27+/+ and p27−/−(Δ51) cells were pre‐treated with MG132 for 12 h and the cells were then incubated with CHX for the indicated times. The cell extracts were subjected to western blots to analyse Jun B protein degradation rates. GAPDH was used as a protein loading control. (F and G) Western blots were employed to compare ITCH protein expression in BEP2D(pTRE‐GFP‐p27) cells treated with or without doxycycline exposure (F), and p27+/+ versus p27−/−(Δ51) cells (G), respectively. GAPDH was used as a protein loading control. (H) The cell extracts from the indicated transfectants were subjected to western blots to evaluate Jun B and JAK1 protein expressions using specific antibodies. β‐Actin were used as protein loading control. (I) The schematic illustration of this study.

Our above results reveal that Jun B is a key regulator in p27/miR‐494/JAK1/STAT3 axis, we evaluated its expression upon NNK exposure in BEP2D cells and in lung tumour tissues. As shown in Figure 6A,B, the expression of Jun B in BEP2D cells was obviously retarded upon NNK exposure, while Jun B protein was barely detectable in lung squamous carcinoma tissues as compared to normal controls. Similarly, it exhibited a comparable expression between adenocarcinoma and corresponding normal controls. Thus, we next explored the mechanism underlying Jun B regulation by p27 (Figure 6C,D). The results showed that Jun B mRNA expression was downregulated in GFP‐p27‐overexpressed BEP2D cells and increased in p27 knockout MEF cells, which is controversial to the protein expression profile, demonstrating that p27 regulation of Jun B occurs at either protein stability or protein translation. To test this hypothesis, CHX was utilized to inhibit new protein synthesis to evaluate Jun B protein degradation rates between p27+/+ and p27−/−(Δ51) cells. As shown in Figure 6E, Jun B degradation rate in p27−/−(Δ51) cells was higher than that in p27+/+ cells, indicating that p27 might act on the stabilization of Jun B protein.

It is reported that ITCH is a E3 ligase for Jun B and could mediate Jun B protein degradation.^29^ Therefore, we determined ITCH protein levels in BEP2D (pTRE‐GFP‐p27) cells with or without doxycycline treatment and p27+/+ versus p27−/−(Δ51) cells. As shown in Figure 6F,G, ectopic GFP‐p27 significantly suppressed ITCH expression in BEP2D cells, while knockout of p27 resulted in ITCH protein upregulation as compared to p27+/+ controls. To further evaluate the contribution of ITCH to Jun B protein degradation, we introduced Myc‐ITCH(WT) and Myc‐ITCH(MUT) which carries a mutation of cysteine to serine at site 822 into p27+/+ cells. As shown by the results of Figure 6H, overexpression of Myc‐ITCH(WT) in p27+/+ cells dramatically inhibited Jun B expression and increased JAK1 protein level, while ectopic expression of Myc‐ITCH (MUT) did not show any observable effect on Jun B protein expression and JAK1 protein level, revealing that ITCH plays an essential role in modulating Jun B protein degradation and thus affecting its downstream JAK1/STAT3 pathway activation. All together, we uncovered a novel pathway of p27 in lung squamous carcinoma carcinogenesis via ITCH/Jun B/miR‐494/JAK1/STAT3 axis.

## 4 DISCUSSION

Previous studies have associated the downregulation of p27 with lung carcinoma development,^30^ However, they predominantly focus on the inhibition of lung carcinogenesis by p27 through its CDK‐dependent function. In this study, we investigated the role of p27 in NNK‐induced lung carcinogenic effect, which has not been explored before. Our results indicated that p27 inhibition of ITCH could reduce Jun B protein degradation and subsequently result in Jun B protein accumulation, which inhibits miR‐494 transcription and expression, thereby reducing its binding to 3′‐UTR of JAK1 mRNA. This leads to a decrease in JAK1 protein expression, and consequently attenuates STAT3 phosphorylation and inhibits the transformation of HBECs. These novel findings provide new insight into understanding the mechanisms of NNK‐induced lung squamous carcinoma as schematic illustration in Figure 6I.

Lung carcinogenesis can largely be attributed to tobacco smoke carcinogens such as the very potent NNK, and the STAT3 has been found to be highly activated in most lung tumour tissues and cell lines. However, the association of STAT3 with the tobacco carcinogen NNK has yet to be explored. Previous studies have discovered that NNK exposure could cause mutations of specific genes such as K‐ras, p53 and EGFR.^31^ In this study, we identified a new pathway whereby NNK induces JAK1 expression by targeting p27. This activation of STAT3 contributes to the transformation of NHBECs. STAT3, originally identified as an acute phase response factor to IL‐6, has been well demonstrated to be associated more frequently than other STATs with tumour formation.^32^, ^33^ A significant body of evidence points to the importance of upstream regulators of STAT3 in lung cancer development, for example, EGFR and Src,^34^ our study highlights JAK1 as a candidate kinase for STAT3 phosphorylation and activation in LUSC. Constitutive activation of STAT3 functions as a transcription factor, inducing downstream target genes that are important for cell proliferation, induction of angiogenesis, prevention of apoptosis, evasion of host immune surveillance or cancer stem cell self‐renewal,^35^ thus further initiating the tumorigenesis.

Our most recent studies show that in human invasive bladder cancer cells, p27 inhibits Calpian1 proteolysis cascade through attenuation of the JAK1/STAT1 axis to stabilize HSP90 protein.^36^ In our current studies, we focused on the detailed mechanisms underlying the p27 inhibition of JAK1, thereby greatly facilitating our understanding of the p27 crosstalk with JAK1/STAT pathway in carcinogenesis. For the first time to the best of our knowledge, we demonstrate that p27 modulates JAK1 mRNA stability through a miR494‐dependent pathway. Our results suggest that the canonical RNA binding proteins do not participate in regulation of JAK1 mRNA stability. Consequently, we turn to miRNAs candidates that might account for JAK1 mRNA alteration. We further noticed that miR‐494 stabilized JAK1 mRNA upon its binding to its 3′‐UTR, unlike the mechanism in which most miRNAs bind to the 3′‐UTR of its targeted mRNA and promotes mRNA degradation or represses protein translation. These novel findings provide new insight into the miRNA functions on their targeted genes.

miRNAs are involved in regulating a number of biological functions including cell cycle, proliferation, differentiation and apoptosis.^37^ miR‐494 is diversely expressed in different cancers and tissues, exerting various functions.^38^, ^39^, ^40^, ^41^ Our laboratory has recently discovered that ChlA‐F treatment significantly induces miR‐494 expression in human invasive bladder cancer cells and the elevated miR‐494 notably attenuated bladder cancer invasion, thus acting as a tumour suppressor.^42^ However, in non‐small cell lung cancer (NSCLC), miR‐494 was reported to participate in lung cancer onset and progression,^43^ while in situ hybridization performed in NSCLC tissues exhibited highly expressed miR‐494, whose high expression level is related to a shorter overall survival in NSCLC patients.^44^ Nevertheless, the underlying mechanisms for its overexpression remain poorly understood. In our study, miR‐494 is highly expressed in lung squamous cell carcinoma but not in adenocarcinoma as compared to their corresponding controls, revealing its potential oncogenic role in human squamous carcinoma. We further discover that Jak1 mRNA acts as a novel target of miR‐494, and leading to Jak1 mRNA stabilization, in turn promoting STAT3 phosphorylation and activation, as well as transformation of NHBECs. Given the important role of miR‐494 in LUSC, miR‐494 might be a candidate for further exploration as a potential biomarker for lung cancer development.

By tracking the upstream pathway involved in miR‐494 alteration regulated by p27, our current studies suggest that Jun B is the transcription factor for negative regulation of miR‐494 transcription, while ITCH, the E3 ligase of Jun B protein, is suppressed by p27. Jun B acting as a transcription factor for directly regulating miR‐494 expression was further supported by ChIP assay using anti‐Jun B antibody, which demonstrated Jun B binding to the specific binding site on the miR‐494 promoter region. Our studies strongly indicate that Jun B decreases upon NNK exposure and is downregulated in lung squamous carcinoma. Here, we verified that Jun B plays a tumour suppressive role in the tumorigenesis of LUSC, and the regulation of itself occurs through its protein degradation modulated by its E3 ligase ITCH‐mediated its protein degradation. ITCH has been previously found to be crucial in the control of the proteasomal degradation of several important substrates involved in the regulation of the programmed cell death pathway and/or the inflammatory responses, including the p53 family members p73 and p63,^45^ the tumour suppressor RASSF5/NORE1,^46^ as well as members of the activator protein 1 (C‐JUN and Jun B).^47^ The catalytic activity of ITCH is directly upregulated by its phosphorylation, which increases its catalytic activity, resulting in Jun B polyubiquitylation.^48^ In our study, the wild type ITCH, and its mutated form that the active site cysteine has been mutated, were both overexpressed in p27+/+ cells. The results indicate that overexpression of ITCH promotes Jun B degradation, whereas the mutated form loses this activity, confirming that ITCH acts as E3 ligase responsible for p27 regulating Jun B protein degradation.

In summary, our studies define an inhibitory effect of p27 on JAK1 expression by suppressing Jun B‐mediated negative regulation of miR‐494 transcription. This leads to a reduction of the direct binding of miR‐494 to the 3′‐UTR region of JAK1 mRNA, thereby resulting in increased JAK1 mRNA degradation. Our studies also show that p27 promotes Jun B protein expression through the inhibition of ITCH expression, which acts as a Jun B E3 ligase, subsequently decreasing ITCH‐dependent Jun B degradation. These novel findings provide significant insights into understanding the underlying mechanisms of p27 in regulation of lung squamous carcinoma development.

The treatment for lung cancer includes surgery, radiotherapy and targeted therapies with antiangiogenic monoclonal antibodies or tyrosine kinase inhibitors (TKIs) for tumours with specific mutations. Although around 60% of NSCLC patients exhibit molecular alterations amenable to targeted therapy, such as mutations in EGFR, ALK, ROS1 and KRAS, the remaining patients, especially those with LUSC (lung squamous cell carcinoma), lack available targets, not to mention the drug resistance associated with current targeted therapies. Consequently, a deeper understanding of the molecular mechanisms underlying lung cancer development is essential. We acknowledge the presence of certain limitations in our study. Initially, the restricted number of tumour tissue specimens available for the experiment might introduce some degree of bias. Furthermore, animal experiments to validate the tumorigenic potential of NNK‐transformed cells in an in vivo model were not performed. Despite these limitations, the findings from our research offer significant insights for the clinical application of ITCH E3 ligase, miR494 or STAT3 as potential targets in the future exploration of lung cancer.

## AUTHOR CONTRIBUTIONS

**Minggang Peng:** Conceptualization (equal); funding acquisition (lead); investigation (lead); writing – original draft (lead); writing – review and editing (lead). **Hao Meng:** Writing – review and editing (equal). **Jingjing Wang:** Data curation (equal); formal analysis (equal); resources (equal). **Mengxin Guo:** Data curation (equal); formal analysis (equal); investigation (equal); software (equal). **Tengda Li:** Investigation (equal); methodology (equal); validation (equal). **Xiaohui Qian:** Data curation (equal); formal analysis (equal); software (equal). **Ruifan Chen:** Data curation (equal); formal analysis (equal); methodology (equal). **Honglei Jin:** Conceptualization (equal); investigation (equal); visualization (equal). **Chuanshu Huang:** Funding acquisition (supporting); project administration (lead); supervision (supporting).

## FUNDING INFORMATION

This work was partially supported by grants from Natural Science Foundation of China (NSFC82002771, NSFC81773391 and NSFC81702530), Oujiang Research Project OJQD2022006 and Discipline Cluster of Oncology, Wenzhou Medical University, China.

## CONFLICT OF INTEREST STATEMENT

The authors report that they have no conflict of interest.

## Supporting information


Figure S1.

## Data Availability

The data generated in this study are available within this article and its supplementary data files. We obtained expression profile data analysed in this study from the HPA and TCGA database.
