# Supplementary material for: p27 specifically decreases in squamous carcinoma, and mediates NNK‐induced transformation of human bronchial epithelial cells
Source: J Cell Mol Med. 2024 Aug 4;28(15):e18577. doi: 10.1111/jcmm.18577 (PMC11298314; doi:10.1111/jcmm.18577)
Supplement: Supplementary file 1 — Figure S1. [file JCMM-28-e18577-s001.pptx]

## Slide 1
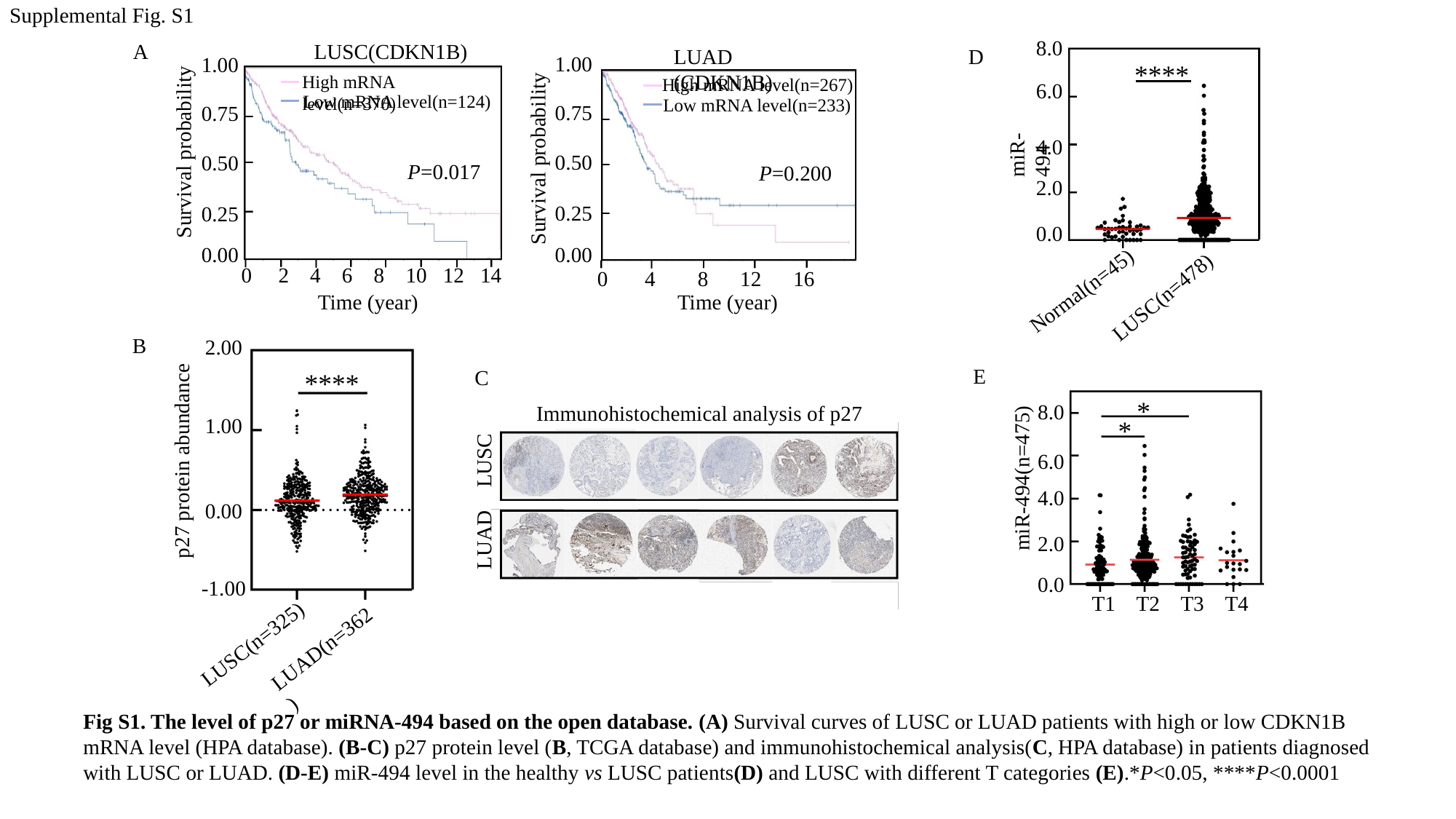

Supplemental Fig. S1
1.00
0.75
0.50
0.25
0.00
1.00
0.75
0.50
0.25
0.00
8.0
6.0
4.0
2.0
0.0
A
LUSC(CDKN1B)
LUAD (CDKN1B)
D
****
High mRNA level(n=370)
High mRNA level(n=267)
Low mRNA level(n=124)
Low mRNA level(n=233)
miR-494
Survival probability
Survival probability
P=0.017
P=0.200
Normal(n=45)
0 2 4 6 8 10 12 14
0 4 8 12 16
LUSC(n=478)
Time (year)
Time (year)
2.00
1.00
0.00
-1.00
B
8.0
6.0
4.0
2.0
0.0
E
C
****
*
LUSC
Immunohistochemical analysis of p27
*
p27 protein abundance
miR-494(n=475)
LUAD
T1 T2 T3 T4
LUSC(n=325)
LUAD(n=362)
Fig S1. The level of p27 or miRNA-494 based on the open database. (A) Survival curves of LUSC or LUAD patients with high or low CDKN1B mRNA level (HPA database). (B-C) p27 protein level (B, TCGA database) and immunohistochemical analysis(C, HPA database) in patients diagnosed with LUSC or LUAD. (D-E) miR-494 level in the healthy vs LUSC patients(D) and LUSC with different T categories (E).*P<0.05, ****P<0.0001
